# Objective and Non-Invasive Evaluation of Fascial Layers Related to Surgical or Post-Traumatic Scars: A Systematic Review of the Literature

**DOI:** 10.3390/life16010133

**Published:** 2026-01-15

**Authors:** Clara De Luca, Yunfeng Sun, Antonio Stecco, Caterina Fede, Claudia Clair, Carmelo Pirri, Giulia Trovarelli, Carla Stecco

**Affiliations:** 1 Institute of Human Anatomy, Department of Neuroscience, University of Padova, 35121 Padova, Italy; 2 Padova Neuroscience Center, University of Padova, 35129 Padova, Italy; 3 Department of Rehabilitation Medicine, NYU Grossman School of Medicine, New York, NY 10016, USA; 4 Department of Orthopedics and Orthopedic Oncology, University of Padua, 35122 Padova, Italy; 5 Department of Surgery Oncology and Gastroenterology (DISCOG), University of Padova, 35122 Padova, Italy

**Keywords:** fascia, wound, scar, ultrasound, shear wave elastography, magnetic resonance imaging, strain elastography, hypodermis, subcutaneous tissue

## Abstract

**Background**: Wound healing contributes to restoring skin integrity. However, scars affect soft tissue in all its layers, including the superficial and deep fascia; moreover, it has been demonstrated that the fibroblasts leading the scarring process develop from progenitors located in the superficial fascia. In the past, research into scar etiology has focused primarily on the dermal and epidermal layers, leaving the role of the fasciae largely overlooked. Many patients presenting with surgical or traumatic scars complain of the increased stiffness and thickness of the scar, reduced extensibility of the area surrounding it, and chronic pain persisting even after the healing process has been completed. The purpose of this systematic review is to investigate the non-invasive tools and methods employed for the objective evaluation of scars that involve fascial layers. **Methods**: A systematic literature search was conducted on PubMed and WOS. Registration DOI: 10.17605/OSF.IO/SDR3Q. **Results:** A total of 11 articles were selected; the etiologies of scars were surgical, traumatic, and other (keloids). The investigations were conducted using ultrasound, magnetic resonance imaging, strain elastography, and shear wave elastography on the visceral fasciae, superficial fascia, hypodermis, and musculoskeletal fasciae. Sliding of fasciae was assessed by ultrasound; thickness of fasciae was assessed by ultrasound and magnetic resonance imaging; stiffness was assessed by shear wave elastography and strain elastography; and the qualitative assessment was performed via ultrasound. **Conclusions:** Our literature review showed that ultrasound, magnetic resonance imaging, strain elastography, and shear wave elastography are currently adopted for investigating the sliding, thickness, stiffness, and qualitative features of scars involving fascial layers. Moreover, our research showed the existence of a gap in the scientific literature on this topic.

## 1. Introduction

The process of wound healing is crucial for the restoration of skin integrity and, therefore, for the maintenance of the barrier that defends the whole body from pathogens coming from the outside [1,2,3,4]. Wound healing in the skin or internal organs is a dynamic process characterized by a precise sequence of coordinated events [5], which may be grouped into four phases: (i) coagulation and hemostasis; (ii) inflammation; (iii) proliferation; and (iv) wound remodeling with scar tissue formation [5,6,7]. However, this process may lead, in some cases, to pathological outcomes, such as chronic wounds, keloids, or hypertrophic scarring [1,8,9,10,11]. Given the rising number of people affected by pathological scarring [9,12], considerable efforts have been put into finding objective and subjective tools for a more comprehensive and precise evaluation of these conditions [4,13,14].

In their article discussing active scars, defined as those producing changes in soft tissues, Lewit and Olsanska stated that “scars affect soft tissue in all its layers from the skin to the subcutaneous tissues, the superficial and deep fascias, the muscles, and even the tissues of the abdominal cavity” [15]. Correa-Gallegos et al., in 2023, introduced a new perspective on the topic by demonstrating that the specialized fibroblasts leading and regulating the skin’s healing process develop from progenitors located in the superficial fascia [16]. This discovery implies that we should not only focus our attention on the epidermis and dermis when managing a scar, but we should also take into consideration the underlying fascial layers, such as the superficial and deep fasciae. According to the most recent definition [17], the fascia system is a “layered body-wide multiscale network of connective tissue that allows tensional loading and shearing mobility along its interfaces”; four organs account for the fascia system: the superficial fascia, musculoskeletal (deep) fascia, visceral fascia, and neural fascia. The superficial fascia divides the subcutaneous tissue (also known as hypodermis) into two different layers, the superficial adipose tissue (SAT) and the deep adipose tissue (DAT); the musculoskeletal fascia is composed of layered networks of connective tissue related to the muscles; the visceral fascia includes layers of connective tissue which allow tensional loading among different organs and viscera and shearing mobility along their interfaces [17,18].

Extensive research has been conducted over the past few decades to define the etiology, pathology, and management of scars affecting the skin [19,20,21,22,23,24,25,26,27,28,29,30,31,32,33,34,35,36,37,38,39]. The prevailing focus of this literature, however, has been on the epidermis and dermis, leading to a paucity of studies investigating the role of the fasciae during the wound healing process.

Many patients presenting with surgical or traumatic scars complain of the increased stiffness and thickness of the scar, the reduced extensibility of the skin surrounding the scar, and chronic pain persisting even after the healing process has been completed [40,41]. Kobesova et al., in 2007 [42], exposed the concept that “skin, fascia, ligaments, and tendons must stretch shift in concert”. In other words, the gliding of one layer must remain independent from the others during the movement. When the process of wound healing leads to the loss of this independence, the scar is defined as adhesive [42,43], and many symptoms related to pathological scars, such as pain and limited range of motion, could be considered a direct consequence of the involvement of fascial layers [2]. Therefore, a more comprehensive evaluation of scar tissue should involve the epidermis, dermis, superficial adipose tissue, superficial fascia, deep adipose tissue, deep fascia, and visceral fascia [2,15].

This systematic review is designed to identify the diagnostic tools employed for the objective assessment of scars affecting the fascial layers, with the purpose of offering clinicians a valuable summary of the diagnostic techniques and the parameters that best evaluate the influence of scarring on fascial integrity. A better knowledge of the tools that may be used to assess the fascia layers will lead in the future to more thorough patient care.

## 2. Methods

### 2.1. Information Sources and Strategy

A systematic literature search was conducted according to the PRISMA-ScR guidelines [44]. We consulted the databases PubMed and Web of Science (WOS). No restriction was imposed on the publication date of the articles. The research was carried out by one reviewer between December 2024 and April 2025, and the same search strategy was adopted for both databases. This systematic review was registered on OSF with registration DOI 10.17605/OSF.IO/SDR3Q.

The following eleven key words were combined in nineteen different ways: fascia, scar, imaging, outcome, assessment, skin, ultrasonography, evaluation, hypodermis, ultrasound, and subcutis. The precise PubMed and WOS entries are listed in Table 1.

### 2.2. Eligibility Criteria

The PICOS model was adopted for developing the search strategy for the present systematic review [45]. We included articles discussing tools and methods used for assessing fascia layers in the site where a scar is located. We searched both imaging and non-imaging techniques applied in vivo on humans, focusing our attention on non-invasive devices, so invasiveness was an exclusion criterion. Studies were also excluded if they were descriptive commentaries. No filter was applied during the research process; no restrictions were added in terms of year of publication; we did not require a minimum number of participants in each study; and scars with any etiology were included in this review. The characteristics we looked for in the articles we reviewed are listed in Table 2.

### 2.3. Definition of Wound

A wound is defined as the disruption of normal anatomic structure and function [6]. This process may involve any organ of the body, and it may begin internally or externally. The deposition of scar tissue is part of the physiological tissue repair process. The depth of the wound influences the size of the scar, which might be minimal if the wound is superficial, or severe or even pathological if the wound is deeper and affects more tissues [46].

Keeping this definition and the purpose of this research in mind, we considered all articles describing the evaluation of wounds located in the skin or in underlying fascial structures to be eligible for the present review.

### 2.4. Study Selection

During the first step of the process, one reviewer screened the article titles found with the literature search for eligibility. In the following step, the eligible articles were screened by reading the abstracts. Finally, two reviewers independently read each one of the selected articles to decide on their inclusion in the review in accordance with the eligibility parameters previously agreed upon. Disagreements about the inclusion of articles were solved through discussion.

### 2.5. Data Charting Process and Synthesis of Results

Once the process of selecting the articles was completed, we summarized the relevant information of each article. We then categorized the articles according to the technology they were describing, and we proceeded with their analysis according to this parameter.

### 2.6. Risk of Bias in Individual Studies

The risk of bias in observational studies was assessed with the ROBINS-E tool (Launch version, June 2022) [47]. The risk of bias in case reports was assessed with the JBI Critical Appraisal Checklist for Case Reports [48].

## 3. Results

### 3.1. Study Selection

**Figure 1:**
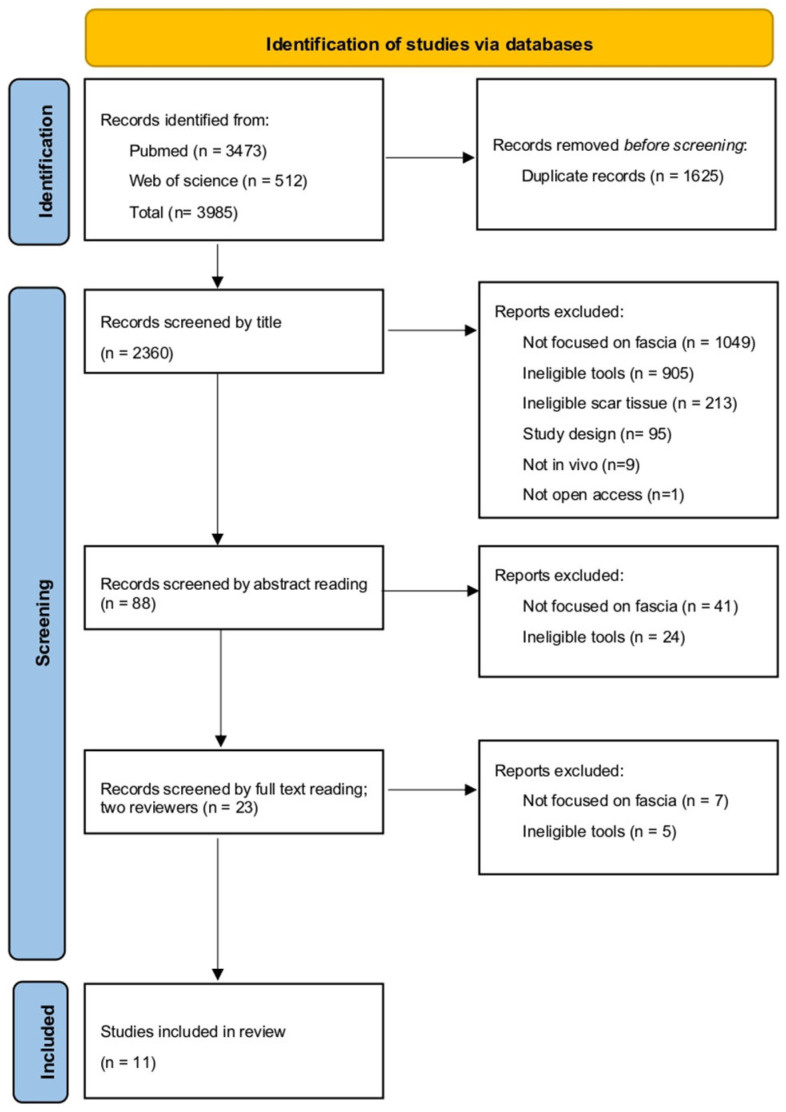
PRISMA 2020 flow diagram of the selection process.

From the research on the literature databases, 3985 results were obtained. After removing the duplicates, one reviewer screened the 2360 articles based on title reading, and 88 articles were selected. Among the excluded articles, 1049 articles were excluded because they did not focus their attention on the fascia system; 905 articles were excluded because the tools described were not pertinent to our criteria—they were not objective methods, they were invasive, or they were simple assessment scales—213 articles were excluded because they did not evaluate scar tissue; 95 articles were excluded because the study design was not pertinent to our criteria; 9 articles were excluded because the analysis conducted were not in vivo; and 1 article was excluded because it was not open access. One reviewer then proceeded to read the abstracts and selected 23 articles; 41 articles were excluded because they did not focus their attention on the fascia system, and 24 articles were excluded because the tools described were not pertinent to our criteria. After reading the full text of the 23 selected articles, two reviewers agreed to consider 11 articles pertinent to our established criteria. The process is illustrated in Figure 1 [49].

### 3.2. Study Characteristics

Table 3 presents the characteristics of the included articles according to the inclusion and exclusion criteria.

### 3.3. Etiology and Location of the Scars

Most of the articles took surgical scars into consideration. In five articles, the surgical procedures were C-sections [50,51,53,57,58]; in one article, the scar was from an orthopedic procedure [60]; and one article discussed general surgeries of the abdomen [61]. On the other hand, three articles considered scars resulting from a previous trauma [52,54,56], and one article evaluated keloids without specifying their etiology [55].

Many areas of the body were taken into consideration: six articles discussed abdominal and pelvic scars [50,51,53,57,58,61]; four articles discussed scars in the limbs [52,54,56,60]; and one article discussed scars affecting the face and trunk [55]. Table 4 shows the regions of the body investigated in each article.

### 3.4. Assessed Tissue

The articles included in this review discussed the visceral fasciae in four cases [50,51,53,58]; the musculoskeletal fascia in three cases [52,54,60]; the hypodermis in four cases [55,56,57,59]; and one article discussing the hypodermis also addressed the superficial fascia [56]. Among the articles discussing the musculoskeletal fascia, two articles discussed the plantar fascia [52,60].

Those articles discussing the visceral fasciae did not always consider the same structures: two articles discussed sliding between the uterus and the abdominal wall [51,53]; one article discussed sliding between the uterine serosa and the fascia transversalis [50]; and one article discussed sliding between the uterus and the abdominal fascia [58]. Table 5 shows the tissues that were investigated in each article.

### 3.5. Included Tools

We selected seven articles discussing ultrasound (US) alone [50,51,52,53,55,58,62], one article discussing a combination of US and strain elastography (SE) [59], two articles discussing shear wave elastography (SWE) [54,57], and one article discussing magnetic resonance imaging (MRI) [60].

**Ultrasound**—We selected eight articles that discussed the US evaluation of fascial layers in a region of the body affected by scar formation. The study design was a case report in three cases [52,56,59], a prospective cohort study in two cases [51,58], a prospective observational double-blinded study in one case [50], a prospective observational blind study in one other case [53], and a retrospective study in one case [55]. All the articles were published between 2017 and 2024. Considering all the articles, 1012 patients were evaluated with a US. Many areas of the body and scar etiologies were considered in this pool of articles: four studies evaluated the utility of US in assessing a C-section scar from a previous pregnancy on pregnant women [50,51,53,58]; one article compared the aspect of surgical scars in the abdomen and stretch marks in the same region [61]; one article evaluated a traumatic scar in the knee [56]; one article evaluated the scar tissue in relation to the plantar fascia after a spontaneous tear [52]; and one article evaluated the aspect of keloids affecting the face and trunk [55]. Pirri et al. visualized the subcutaneous tissue and the superficial fascia in the knee region using a US; moreover, they emphasized the concept of sonopalpation—a technique that utilizes the probe for precise identification of painful spots. According to their experience, US with sonopalpation is a useful tool for follow-up with patients with scars, because it is objective and it takes into consideration the generation of pain. Cocco et al. examined two plantar fasciae after a partial and a complete tear. With this study, they showed that US is a valid instrument for the investigation of the deep fascia when its continuity is compromised. US was able to discriminate between the fascial tissue and the fibrotic tissue resulting from the scarring process. Veronese et al. showed that US was able to detect the thickening of the dermis–hypodermis transition and thickening of the hypodermis in the region affected by a scar; this same aspect was also found by Pirri et al., who identified the retinacula cutis as the reason for this thickening. Lobos et al. identified the involvement of the hypodermis in 5% of the keloids they analyzed; therefore, they also show that US is a valid tool for the assessment of this layer. Baron et al., Charernjiratragul et al., Drukker et al., and Sönmez et al. investigated the accuracy of the sliding sign in predicting the risk of uterine adhesions in pregnant women with a history of a previous C-section. All of these authors agreed that a US could be considered an appropriate tool to investigate the presence of adhesions between the uterus and the surrounding organs.

**Shear wave elastography**—We selected two articles that focused on the use of shear wave elastography (SWE) for the evaluation of fasciae affected by a scarring process. One article was designed as an observational study [54], and the other one was a prospective cross-sectional study [57]. The first was published in 2020, the latter in 2021. Kawai et al. included 11 participants in their study; Seven et al. included 102 participants, resulting in 113 patients in total. These articles took into consideration two different tissues: Kawai et al. analyzed the musculoskeletal fascia located superficially to the hamstring following a hamstring strain; Seven et al. analyzed the subcutaneous tissue stiffness in relation to a previous C-section scar. Kawai et al. found a statistically significant difference in stiffness between the musculoskeletal fascia of the injured limb and the contralateral; Seven et al. found that SWE was able to predict whether a C-section scar would present adhesions by considering its stiffness grade. The hypothesis, which was in fact validated, was that stiffer layers in the subcutis would indicate a higher presence of adhesions.

**Strain elastography**—We selected one article discussing the use of SE for the assessment of the hypodermis in the region of a scar. This case report by Veronese et al. was published in 2024; it evaluated a scar located on the abdomen and compared it to intact skin and stretch marks located in the same area. In the article by Veronese et al., SE showed increased stiffness in stretch marks and scar tissue compared to intact skin, but only in the longitudinal direction. Moreover, the structural components of intact skin were different compared to those of the stretch marks and scar tissue in a statistically significant way.

**Magnetic resonance imaging**—We included one article in our review that discussed the use of MRI for the visualization and analysis of the fascial system. This retrospective study by Yu et al. was published in 1999 and discussed plantar fasciotomy; 15 patients were included. MRI was chosen for the description of the post-op appearance of the plantar fascia; its thickness was found to be doubled at the site of the fasciotomy compared to the contralateral side.

### 3.6. Outcomes of the Articles

We included articles in our review that presented the assessment of the thickness of the fascial layers, sliding between the fascial layers, the stiffness of the fascial layers, or a qualitative assessment, such as a simple visualization of the fasciae, among their outcomes.

Thickness was assessed in three articles [56,59,60]; sliding was assessed in four articles [50,51,53,58]; stiffness was assessed in three articles [54,57,59]; and four articles performed a qualitative assessment of the fasciae [52,55,56,59] (Table 6).

**Thickness**—The case report by Pirri et al. investigated the subcutaneous tissue lying underneath a traumatic scar located on the knee via US. The authors reported the thickening of the subcutaneous tissue (2.33 mm) compared to the contralateral side (1.31 mm). Veronese et al. investigated the hypodermis lying underneath a surgical scar and a stretch mark via US. They observed thick connective fibers in the hypodermis near the dermal-hypodermal junction. Yu et al. measured the thickness of the plantar fascia after fasciotomy using MRI. They observed that the thickness of the plantar fascia after surgery was nearly double that of its pre-operative measurement; however, no further data were provided on this topic. None of these articles produced statistically significant results.

**Sliding**—Four articles aimed at evaluating the reliability of the sliding sign in a gynecological context. This is a sonographic sign meant to detect any existing gliding plane between the abdominal or pelvic organs and the fascia transversalis or the abdominal fascia. The absence of gliding is explained by the existence of adhesions connecting the different layers and limiting the independence of each layer from its surroundings. The sign is defined as positive if sliding is detected and negative if, on the contrary, no sliding is visible. These studies were designed to investigate the reliability of the sliding sign by finding the correlation between a negative sliding sign and the presence of adhesions, which was assessed during a subsequent C-section performed on the same patient [50,51,53,58]. Baron et al. [50] investigated whether US is an adequate tool for the detection of adhesions between the uterine serosa muscle fascia and the fascia transversalis in pregnant women who had undergone a past C-section. They investigated the presence of the sliding sign during the execution of deep breaths with trans-abdominal ultrasound (TAS): a negative sliding sign, suggesting the presence of adhesions, was found in 19 patients and confirmed during surgery in 16 patients; a positive sliding sign, suggesting free gliding and therefore the absence of adhesions, was found in 40 patients and confirmed during surgery in 35 patients. They concluded that the use of US for assessing the presence of adhesions between the uterus and the abdominal wall has a sensitivity of 76.5% and a specificity of 92.1%; therefore, their data showed that TAS is an adequate tool for discriminating between a low and high risk of intra-abdominal adhesions in pregnant women who had undergone a C-section in the past. Charernjiratragul et al. [51] investigated whether ultrasound is an effective tool for identifying adhesions between the anterior uterine wall and the abdominal wall; they demonstrated that TAS used for this purpose has acceptable sensitivity (39.1%) and high specificity (95.2%) for the detection of adhesions of any severity in this location; moreover, a negative sliding sign can predict the formation of moderate to thick adhesions with an odds ratio of 21.0. Drukker et al. [53] investigated the correlation between a negative sliding sign and the presence of severe adhesions between the anterior uterine wall and the abdominal wall as their primary outcome; they demonstrated a sensitivity of 56%, a specificity of 95%, a positive likelihood ratio of 12.1, and a negative likelihood ratio of 0.46 for the ability to detect severe adhesions. Sönmez et al. [58] evaluated the efficacy of US for detecting adhesions between the uterus and abdominal fascia; they found a statistically significant association between a negative sliding sign at the TAS during pregnancy and the detection of adhesions during the subsequent C-section procedure. In fact, a negative sliding sign had a likelihood ratio of 4.198 for the detection of intra-abdominal adhesions. These results are summarized in Table 7.

**Stiffness**—Kawai et al. investigated the stiffness of fascial tissue covering the hamstring muscle after a strain injury and compared it to the contralateral. They observed that the injured side was significantly stiffer compared to the uninjured side. Seven et al. investigated the subcutaneous tissue stiffness in a previous cesarean incision scar. Pre-operative stiffness was assessed via SWE, followed by a blinded intra-operative grading of adhesions by a senior surgeon during the cesarean delivery; they found that the total adhesion score was statistically higher in the moderate and severe adhesion group compared to the non-adhesion and mild adhesion group. Veronese et al. measured the stiffness of a stretch mark and of a scar. Longitudinally, they found that the stiffness rates were increased compared to an area of intact skin; transversally, intact skin had the highest stiffness level, while scar tissue and stretch marks were more elastic. These results are summarized in Table 8 and Table 9.

**Qualitative assessment**—Cocco et al. performed a qualitative evaluation of the plantar fascia after a traumatic injury. They described a small fibrous bridge between the two stumps of the torn plantar fascia and fibrous scar tissue in the case of a partial tear of the plantar fascia. Lobos et al. investigated the US appearance of keloids, finding that the hypodermis was involved in 5% cases. Pirri et al. measured not only the thickness of the hypodermis but also observed a hyperechoic aspect of that same layer. Veronese et al. observed that the dermal layer involved in keloids protruded into the hypodermis. As these were qualitative observations, no statistically significant result was obtained.

### 3.7. Risk of Bias in Individual Studies

Regarding the bias of all selected studies, Table 10 summarizes the risk of bias in the case reports, and Table 11 summarizes the risk of bias in the observational studies.

## 4. Discussion

The objective of this systematic review was to identify the tools, methods, and parameters utilized for the objective evaluation of scars involving fascial layers; specifically, we aimed to provide clinicians with a comprehensive overview of the diagnostic techniques used to assess scars’ impact on the fascial layers. We found that US, MRI, SE, and SWE may be effectively utilized for this purpose; the primary outcomes evaluated include the gliding, thickness, and stiffness of the fascial layers affected by scarring. Furthermore, our review identified a gap in the existing scientific literature regarding the appropriate tools for assessing fasciae affected by scar formation.

Eleven terms were included in our search strategy and associated in nineteen different ways; the rationale for selecting such an extensive range of terms is as follows: first, we were not able to find many articles matching our criteria and we wanted to make sure that, by making our field of research as broad as possible, we could include the majority of articles discussing the topic of our interest; second, despite our review being focused on the fascia system, we decided to also include in our research the terms “hypodermis” and “subcutis”, prompted by the lack of terminological consensus regarding this topic.

Most of the included articles discussed scars resulting from surgical procedures, and most of them were located on the uterus: C-section scars were the main topic in five of the eleven articles.

### 4.1. Considered Tissue

**Visceral fascia**—Because of the ambiguity and the lack of consensus around the definition of the fascia system in the literature, we carefully underlined the tissue that was being analyzed in each one of the included articles. Reading the articles discussing C-sections, we found heterogeneity in the nomenclature. In fact, despite all the articles discussing the sliding sign and all of them assessing it the same way, they stated that they were evaluating slightly different structures: two articles were less accurate in the definition of the structures, and they did not mention the fascial layers implicated in the movement [51,53]; two articles took into consideration the sliding plane between the uterus and the abdominal fascia [57,58]; and one article discussed the uterine serosa muscle fascia and the fascia transversalis [50].

**Superficial fascia**—The superficial fascia is located in the hypodermis, and sometimes these two terms are referred to interchangeably. The hypodermis is a fatty layer bordered superiorly by the dermis and inferiorly by the musculoskeletal (deep) fascia [63,64,65]; it could also be referred to as the subcutis, and it is divided into the superficial adipose tissue (SAT) and deep adipose tissue (DAT) by the superficial fascia. Two articles discussed the hypodermis [55,59] and one article discussed the subcutis and the superficial fascia [56].

**Musculoskeletal fascia**—One article discussed fascial tissue, meaning the muscle fascia [54], and two articles discussed the plantar fascia [52,60].

After a careful analysis of the anatomy described in the literature, it is clear that many articles were discussing the same structures (superficial fascia, musculoskeletal fascia, visceral fascia), but they were referring to them with different terms. As stated by Stecco et al. [17], when the same anatomical structures are referred to with different terminology in different articles, it is difficult to synthesize findings and to carry out comparisons.

We must point out that the present review aimed to report on the state of the art concerning the evaluation of fascial structures involved in scarring in any body district. Therefore, we included articles discussing scars located in multiple anatomical sites, being aware that the influence of a scar on the surrounding tissues depends greatly on the anatomical site itself. In fact, the involvement of the fascial system in the process of scar formation is determined firstly by its anatomical and functional characteristics: for instance, the layer of adipose tissue interposed between the superficial fascia and musculoskeletal fascia is much more pronounced in the abdominal wall compared to the hamstring region, and this influences not only the anatomical aspect of the scar but also its dynamic outcome. With our work, we did not intend to compare scars located in different body regions, but we aimed to offer the reader a comprehensive survey of the current knowledge on the topic.

### 4.2. Included Tools

US was the most investigated diagnostic tool in this review. Many factors could explain this: first, most of the time it was adopted for the assessment of C-section scars in pregnant women, and this is the routine exam for this population, given its safety and availability; moreover, this method is the frequent choice for the assessment of superficial structures, such as the dermis or the hypodermis, thanks to the good accuracy and precision that it offers and its low risk for the patients’ health [66,67,68]. This allowed us to obtain information about the quantitative and qualitative analysis of the tissues of interest and the presence of adhesions. The articles discussing SWE indicated whether the considered tissue had a higher grade of stiffness compared to the controls. SE was adopted in one article for the assessment of stiffness. Only one article discussed MRI; this tool, like US, showed the qualitative structure of the tissues.

**Ultrasound**—This tool has an important application in the obstetric–gynaecologic field, as the presence of a free gliding plane between the uterus and the abdominal fascia is interpreted as the absence of adherences resulting from a previous C-section. These results indicate that ultrasound (US) is a reliable tool for examining fascial layers in the scarring process, providing both precise and accurate data. Future research should expand the investigation of the sliding sign to additional anatomical regions; furthermore, significant effort must be directed toward establishing standardized protocols that enable the quantitative analysis of the sliding sign. Many articles showed that US is a valid tool for visualizing the different layers and fasciae of the subcutaneous tissue; moreover, abnormalities may be investigated with this tool, both in a qualitative way, by looking at the echogenicity or searching for the sliding sign, and in a quantitative way, by measuring the thickness of the layers. More precisely, US was successfully adopted for the evaluation of the hypodermis and superficial fascia [55,56,61], musculoskeletal fascia [52], and visceral fascia [50,51,53,58]. Pain and stiffness associated with surgical or traumatic scars represent common clinical complaints; therefore, future large-scale studies should evaluate the clinical utility of sonopalpation, as introduced by Pirri et al., within this diagnostic context. While standard US facilitates the qualitative visualization of anatomical structures, the integration of quantitative measurements will provide a more rigorous assessment. Furthermore, the application of sonopalpation will yield critical functional data, clarifying the relationship between clinical symptoms and sonographic findings.

**Shear wave elastography and strain elastography**—SWE and SE were successfully employed for the investigation of the hypodermis [57,59] and musculoskeletal fascia [54]. In all cases, the authors identified a local stiffness in the region affected by a scar. Kawai et al. [54] demonstrated a promising application of SWE: they were the first group to observe an increased stiffness in the fascial tissue in the hamstring region after a strain injury compared to the contralateral. Given that increased fibrotic tissue and reduced elasticity within the fascia negatively impact the biomechanical dynamics of the muscle, this assessment tool is highly suitable for evaluating athletes following muscle injuries before they return to their training. Future investigations should focus on the stiffness of soft tissues and fasciae associated with scarring across diverse anatomical regions; such research holds significant clinical and functional value, as it could elucidate whether persistent musculoskeletal fascial rigidity following muscle strain contributes to chronic muscular dysfunction. In the future, SE should be replaced by SWE because strain elastography is less accurate than SWE, and the results are more dependent on the operator.

**Magnetic resonance imaging**—The article by Yu et al. showed that MRI is an accurate tool for visualizing the plantar fascia and distinguishing between a normal state and a thickened one; however, few pre-operative images were available in the article, therefore more research should be carried out on this topic in the future for a better understanding of how to employ this tool for the analysis of the involvement of fasciae in scar formation.

### 4.3. Outcomes of the Articles

**Thickness**—While thickening of the skin and dermis is well-documented in hypertrophic scars and keloids [69,70], fascial layers—such as the superficial and deep fascia—have been investigated in far fewer studies. Thickness was measured in three articles by US and MRI. Both of these tools allow the precise measurement of the thickness of layers; however, these articles had a limited sample size, and none of them could produce statistically significant results; therefore, more data should be collected to better investigate the application of US and MRI in measuring the thickness of fasciae. In general, US is faster, cheaper, and more indicated for a close follow-up, compared to MRI. Measuring the thickness of fascial structures is crucial as it is an indirect indicator of fibrosis. In fact, the deposition of collagen and other fibers on the fascial layer caused by a pro-fibrotic environment, such as the wound, leads to thickening of the fascial layer.

**Sliding**—According to the four articles included in this review, the sliding sign in a gynecologic context measured with US is a reliable method for assessing the presence of adhesions [50,51,53,58,71]. The totality of these articles produced statistically significant results supporting the accuracy of US in identifying adhesions between the uterus and the surrounding fasciae. Even though it appears that these articles investigated slightly different tissues, we should consider that they were all focused on the same organ (uterus) and on the fascial structures of the same district (abdomen and pelvis); therefore, they were likely considering the same tissues, but they were referring to them with different terminology. In fact, the procedure was conducted following the same protocol in all the studies, and the probe was placed at the same level to visualize the interface between the uterus and the surrounding fasciae. While the US enables the visualization of gliding, it does not support a quantitative analysis of this parameter; consequently, observations are limited to identifying whether gliding is present. In the future, more studies should be conducted to understand how to quantify the gliding; moreover, it would be interesting to investigate fascial gliding in other anatomical regions in response to localized movement.

**Stiffness**—While skin and dermal stiffness are well-characterized in hypertrophic scars and keloids [69,70], significantly fewer studies have investigated these properties in fascial structures, such as the superficial and deep fascia. We found three articles investigating the stiffness of fasciae located in several body districts. Stiffness of fascial tissue may be caused by the deposition of fibrotic fibers and a relative reduction in elastic fibers. The presence of a pro-inflammatory environment may be responsible for this activity in the context of wound healing. In this process, the deposition of fibers is meant to restore tissue continuity, but it eventually results in harm to the organism if the process is not carefully regulated. All these articles showed that SWE is able to identify properly a stiffened fascia [54,57]; two of these articles produced statistically significant results, while the other article investigated this topic on a single patient [59]. The clinical significance of fascial stiffness warrants further investigation; during ultrasound assessment, a stiff fascia may appear hyperechoic and thickened, which typically indicates a high degree of fibrosis. Moreover, in the future, it would be interesting to relate the pathologic aspects of fasciae to the clinical presentation of the patients; in fact, pain and limited ROM are frequent symptoms that may be associated with these presentations. As it was underlined by Kawai et al., an elevated fascial stiffness affects the movement and the transmission of forces between the different tissues involved in the execution of motion [54].

**Qualitative assessment**—The qualitative assessment of fasciae may be useful for comparing the echogenicity of two structures. For instance, a more hyperechoic subcutis may be the result of a fibrotic process. A qualitative assessment may also be adopted for investigating the presence of anomalous structures, such as fibrous bridges between the two sides of a torn plantar fascia. The four articles we discussed showed that US and MRI may be employed for the assessment of the qualitative appearance of fasciae; however, more studies should be carried out to define a standardized evaluation protocol.

### 4.4. Limitations

We were able to collect a limited number of articles for the present review. Most of the observational studies, according to the ROBINS-E, resulted in a high or very high risk of bias; given the limited body of evidence currently available on this topic, we chose to include all identified articles regardless of their risk of bias; this inclusive approach was necessary to gather the maximum amount of information possible for our analysis. A significant portion of the included literature consists of case reports characterized by small sample sizes; consequently, most of these studies lack the statistical power to demonstrate significant results. Overall, while the included articles investigated a variety of anatomical sites, data remains sparse for most regions, except for the abdominal-pelvic area. The lack of consensus on the terminology concerning the fascia system may cause difficulty in confronting the results of different articles, as in some cases, it appears as if the same anatomical structures are referred to with different terms.

## 5. Conclusions

In conclusion, our literature review showed that the tools adopted so far for the objective and non-invasive assessment of fascial layers affected by scar formation are US, MRI, SE, and SWE. The thickness and stiffness of the fascial layers indirectly show the tissue composition, as elevated values may be due to fibrotic deposition. Sliding between the fascial layer and the adjacent tissues indicates whether pathological adhesions are present. Furthermore, our research identified a significant gap in the current scientific literature regarding the specific involvement of fasciae during scar formation. Numerous authors have detailed the implications of fascial stiffness, thickening, and adhesions; these pathological changes restrict the free movement of the fasciae, leading to significant alterations in global body mechanics.

Identifying the optimal strategy for assessing fasciae affected by scar tissue formation is essential for delivering comprehensive patient care. In the future, implementing regular fascial assessments following surgical procedures or traumatic events will enable clinicians to detect pathological remodeling patterns at an early stage, thereby facilitating interventions to mitigate their impact on fascial homeostasis.

## Figures and Tables

**Table 1:** PubMed and Web of Science entries.

Research Term	Database Entries
Fascia	“fascia” OR “fasciae” OR “fascias”
Scar	“cicatrix” OR “cicatrix” OR “scar”
Imaging	“image” OR “images” OR “imaging” OR “imagings”
Outcome	“outcome” OR “outcomes”
Assessment	“assess” OR “assessed” OR “assessing” OR “assessment” OR “assessments”
Skin	“skin” [MeSH Terms] OR “skin” [All Fields]
Ultrasonography	“diagnostic imaging” [MeSH Subheading] OR (“diagnostic” AND “imaging”) OR “diagnostic imaging” OR “ultrasonography” OR “ultrasonography” [MeSH Terms]
Evaluation	“evaluability” OR “evaluate” OR “evaluated” OR “evaluates” OR “evaluating” OR “evaluation” OR “evaluations” OR “evaluative” OR “evaluator” OR “evaluators”
Hypodermis	“subcutaneous tissue” [MeSH Terms] OR (“subcutaneous” AND “tissue”) OR “subcutaneous tissue” OR “hypodermis”
Ultrasound	“diagnostic imaging” [MeSH Subheading] OR (“diagnostic” AND “imaging”) OR “diagnostic imaging” [All Fields] OR “ultrasound” [All Fields] OR “ultrasonography” [MeSH Terms] OR “ultrasonography” OR “ultrasonics” [MeSH Terms] OR “ultrasonics” OR “ultrasounds”
Subcutis	“subcutaneous tissue” [MeSH Terms] OR (“subcutaneous” [All Fields] AND “tissue” [All Fields]) OR “subcutaneous tissue” [All Fields] OR “subcutis” [All Fields] OR “subcutaneous fat” [MeSH Terms] OR (“subcutaneous” [All Fields] AND “fat” [All Fields]) OR “subcutaneous fat” [All Fields])

**Table 2:** Inclusion and exclusion criteria according to the PICOS model.

Characteristics	Inclusion	Exclusion
**Participants**	Human participants, in vivo only	Cadavers, animals, or other models; individuals affected by neoplasms.
**Intervention**	Objective diagnostic technique is applied to assess the fascia at the site where a scar was formed for trauma or surgery.	Invasive assessing methods; in vitro analysis.
**Control/Comparator**	No control/comparator was included	
**Outcome measures**	Studies that qualitatively assessed the fascia layers, including the gliding of the fascia at the site of a scar; Studies that quantitatively assessed fascia layers, including thickness, stiffness of a scar.	Subjective reports
**Study design**	Trials, prospective cohort studies, prospective observational studies, case reports, reliability and validity studies, prospective cross-sectional studies, retrospective studies.	Descriptive commentaries, reviews
**Publication**	English; full text available.	Conference abstracts without full data, non-peer-reviewed articles, and non-original papers

**Table 3:** Characteristics extracted from each article.

Study	Participants	Intervention	Control	Outcome Measures	Study Design	Publication
Baron et al. [50]	Humans, in vivo	Ultrasound	No	Sliding	Prospective observational double-blinded study	English language, full text available
Charernjiratragul et al. [51]	Humans, in vivo	Ultrasound	No	Sliding	Prospective cohort study	English language, full text available
Cocco et al. [52]	Humans, in vivo	Ultrasound	No	Qualitative analysis	Case report	English language, full text available
Drukker et al. [53]	Humans, in vivo	Ultrasound	No	Sliding	Prospective blind observational study	English language, full text available
Kawai et al. [54]	Humans, in vivo	Shear wave elastography	No	Stiffness	Observational study	English language, full text available
Lobos et al. [55]	Humans, in vivo	Ultrasound	No	Qualitative analysis	Retrospective study	English language, full text available
Pirri et al. [56]	Humans, in vivo	Ultrasound	No	Thickness, qualitative analysis	Case report	English language, full text available
Seven et al. [57]	Humans, in vivo	Shear wave elastography	No	Stiffness	prospective cross-sectional study	English language, full text available
Sönmez et al. [58]	Humans, in vivo	Ultrasound	No	Sliding	prospective cohort study	English language, full text available
Veronese et al. [59]	Humans, in vivo	Ultrasound, Strain elastography	No	Stiffness, thickness, qualitative analysis	Case report	English language, full text available
Yu et al. [60]	Humans, in vivo	Magnetic resonance imaging	No	Thickness	Observational study	English language, full text available

**Table 4:** Etiology and location of the scars.

	Etiology					Location		
	C-Section	Orthopedic Surgery	General Surgery	Trauma	Keloids	Abdomen and Pelvis	Limbs	Face and Trunk
Baron et al. [50]	X					X		
Charernjiratragul et al. [51]	X					X		
Cocco et al. [52]				X			X	
Drukker et al. [53]	X					X		
Kawai et al. [54]				X			X	
Lobos et al. [55]					X			X
Pirri et al. [56]				X			X	
Seven et al. [57]	X					X		
Sönmez et al. [58]	X					X		
Veronese et al. [59]			X			X		
Yu et al. [60]		X					X	

**Table 5:** Assessed tissue.

	Visceral Fascia	Musculoskeletal Fascia	Hypodermis	Superficial Fascia
Baron et al. [50]	X			
Charernjiratragul et al. [51]	X			
Cocco et al. [52]		X		
Drukker et al. [53]	X			
Kawai et al. [54]		X		
Lobos et al. [55]			X	
Pirri et al. [56]			X	X
Seven et al. [57]			X	
Sönmez et al. [58]	X			
Veronese et al. [59]			X	
Yu et al. [60]		X		

**Table 6:** Outcomes of the articles.

	Thickness	Sliding	Stiffness	Qualitative Assessment
Baron et al. [50]		X		
Charernjiratragul et al. [51]		X		
Cocco et al. [52]				X
Drukker et al. [53]		X		
Kawai et al. [54]			X	
Lobos et al. [55]				X
Pirri et al. [56]	X			X
Seven et al. [57]			X	
Sönmez et al. [58]		X		
Veronese et al. [59]	X		X	X
Yu et al. [60]	X			

**Table 7:** Results concerning the sliding sign.

	Sensibility	Specificity	Positive Likelihood Ratio	Negative Likelihood Ratio
Baron et al. [50]	76.5%	92.1%		
Charernjiratragul et al. [51]	39.1%	95.2%		
Drukker et al. [53]	56.0%	95.0%	12.00	0.46
Sönmez et al. [58]			4.198	

**Table 8:** Results concerning stiffness (part 1).

		Mean Shear Modulus (kPa)	*p* Value
Kawai et al. [54]	Injured Uninjured	17.37 12.70	0.48
Seven et al. [57]	Moderate and severe adhesions Mild adhesions	51.50 36.80	0.003

**Table 9:** Results concerning stiffness (part 2).

		Stiffness Scar (%)	Stiffness Stretchmark (%)	Stiffness Intact Skin (%)
Veronese et al. [59]	Longitudinal Transversal	59.30 58.23	59.44 47.20	44.04 60.79

**Table 10:** Risk of bias in case reports.

	Pirri et al. [56]	Cocco et al. [52]	Veronese et al. [59]
Were the patient’s demographic characteristics clearly described? (yes/no/N.A.)	Yes	Yes	Yes
Was the patient’s history clearly described and presented as a timeline? (yes/no/N.A.)	Yes	Yes	No
Was the current clinical condition of the patient on presentation clearly described? (yes/no/N.A.)	Yes	Yes	Yes
Were diagnostic tests or assessment methods and the results clearly described? (yes/no/N.A.)	Yes	Yes	Yes
Were the intervention(s) or treatment procedure(s) clearly described? (yes/no/N.A.)	N.A.	N.A.	N.A.
Was the post-intervention clinical condition clearly described? (yes/no/N.A.)	N.A.	N.A.	N.A.
Were adverse events (harms) or unanticipated events identified and described? (yes/no/N.A.)	N.A.	N.A.	N.A.
Does the case report provide takeaway lessons? (yes/no/N.A.)	Yes	Yes	Yes

**Table 11:** Risk of bias of observational studies.

	Domain 1 Var A	Domain 2 Var A	Domain 3	Domain 4	Domain 5	Domain 6	Domain 7	Overall Risk of Bias
Baron et al. [50]	●	●	●	●	●	●	●	●
Charernjiratragul et al. [51]	●	●	●	●	●	●	●	●
Drukker et al. [53]	●	●	●	●	●	●	●	●
Kawai et al. [54]	●	●	●	●	●	●	●	●
Lobos et al. [55]	●	●	●	●	●	●	●	●
Seven et al. [57]	●	●	●	●	●	●	●	●
Sönmez et al. [58]	●	●	●	●	●	●	●	●
Yu et al. [60]	●	●	●	●	●	●	●	●

●Low risk of bias; ●Low risk of bias, except for concerns about residual confounding; ●Some concerns; ●High risk of bias; ●Very high risk of bias.

## Data Availability

No new data were created or analyzed in this study.
